# Antagonism-driven upregulation of the pulcherriminic acid biosynthetic pathway in *Metschnikowia pulcherrima* and *Leptosphaeria maculans* interaction

**DOI:** 10.1007/s11274-026-05168-6

**Published:** 2026-08-04

**Authors:** Monika Borkowska, Agnieszka Wita, Kamila Myszka

**Affiliations:** Department of Biotechnology and Food Microbiology, Poznan University of Life Sciences, Wojska Polskiego 48, Poznan, 60-637 Poland

**Keywords:** Antagonistic yeast, Biocontrol agent, Brassica stem canker, Iron sequestration, Gene expression, PUL cluster

## Abstract

*Metschnikowia pulcherrima* is a promising biological control agent (BCA), particularly intended for postharvest fruit protection. The research presents pulcherriminic acid (PA) producing isolates obtained from organic fruit surfaces that are antagonistic towards *Leptosphaeria maculans*, the causal agent of phoma stem canker. A highly antagonistic strain identified as *M. pulcherrima* was selected for studying the interactions with *L. maculans* in various in vitro culture techniques. In solid co-cultures, increased iron bioavailability restricted PA diffusion in the yeast colony microenvironment, significantly reducing antagonism. Double-plate assays indicated that volatile organic compounds (VOCs) contribute to the total inhibitory effect. When evaluating liquid co-cultures, complete pathogen inhibition was achieved within the tested yeast concentrations. The employed culture system simultaneously promoted yeast competitive advantage and allowed for the monitoring of expression changes within the *PUL* gene cluster. Pathogen presence induced a significant sequential expression pattern within the *PUL* gene cluster: an early strong upregulation of *PUL2* encoding PA biosynthesis enzyme and *PUL3* encoding the PA and pulcherrimin transporter, followed by sustained increased expression of *PUL3*, and finally very strong induction of *PUL4*. No changes were observed in *SNF2* expression in co-culture. This model elucidates the molecular mechanisms of yeast antagonism induction, which is essential for developing effective, sustainable plant formulations for preharvest and postharvest protection.

**Supplementary Information:**

The online version contains supplementary material available at 10.1007/s11274-026-05168-6.

## Introduction

*Metschnikowia pulcherrima* is globally distributed and comprises species mainly isolated from the phyllosphere, in particular from flowers and fruits (Horváth et al. 2021; Janakiev et al. 2022; Pawlikowska et al. 2019; Sipiczki 2006; Tatay-Núñez et al. 2024) and additionally, isolated from the digestive tract or frass of some insects, mainly from *Apis mellifera* (Agarbati et al. 2024; Lapinskaitė et al. 2025; Yoshihashi and Degawa 2023), as well as aquatic organisms and marine samples, including the extreme environment of Antarctica (Machado Drumond de Souza et al. 2023; Martins Godinho et al. 2019; Wentzel et al. 2019). It is worth noting that some species of the *Metschnikowia* genus are globally distributed, while others exhibit extreme endemism (Raquel de Oliveira Santos et al. 2025; Khammeankea et al. 2026). The distinctiveness of *Metschnikowia* species extends beyond their ecological diversity to include notable morphological and physiological traits. Comprehensive analyses of primary and secondary barcode sequences have revealed that species within this genus cannot be reliably delimited using traditional species concepts. Furthermore, no clear correlation has been found between genetic and phenotypic diversities. Based on these findings, Sipiczki (2022) suggested merging the members of the *M. pulcherrima* clade into a single species, *M. pulcherrima*, as the definitive taxonomic designation (Sipiczki 2022; Sipiczki et al. 2024). These taxonomic properties were further supported by comparative genomic analyses of *Metschnikowia* isolates, which revealed the absence of single-copy markers for intraclade differentiation (Larini et al. 2025; Troiano et al. 2023).

The regulatory status of *Metschnikowia* species is well-established globally. While EFSA (2017) conducted a peer-reviewed risk assessment for *M. fructicola* NRRL Y-27,328 as a pesticide active substance in the EU, the US FDA (2022) has granted Generally Recognized As Safe (GRAS) status to both *M. pulcherrima* and *M. fructicola.* According to recent scientific reports, none of the investigated *Metschnikowia* strains demonstrated the capacity to produce biogenic amines (Larini et al. 2025). Toxicological evaluations of *M. pulcherrima* KIOM G15050 (formerly *M. persimmonesis* KIOM G15050) extracts revealed no adverse effects on the liver or mitochondria in zebrafish models, nor was any potential cardiotoxicity observed (Rahmat et al. 2024). Furthermore, other *M. pulcherrima* strains (formerly *M. ziziphicola*) selected for their probiotic potential exhibited no hemolytic activity (Staniszewski and Kordowska-Wiater 2023).

*Metschnikowia* spp. exhibited potent inhibitory activity against prevalent phytopathogens, including *Alternaria* spp. (Pawlikowska et al. 2019; Pawlikowska and Kręgiel 2017; Steglińska et al. 2022), *Botrytis cinerea* (Fernandez-San Millan et al. 2023; Lombardo et al. 2023; Pawlikowska et al. 2019; Sipiczki 2006), *Penicillium* spp. (Parafati et al. 2016; Pawlikowska and Kręgiel 2017; Settier-Ramírez et al. 2021; Türkel et al. 2014; Wang et al. 2023). Notably, *Metschnikowia* species exhibit significant antagonism toward *Fusarium* spp., a group of globally distributed pathogens causing fusarioses (Pawlikowska et al. 2019; Rahmat et al. 2024; Steglińska et al. 2022). This antimicrobial potential extends to diverse specialised pathogens, such as *Neofabraea vagabunda* (apple Neofabraea rot) (Bühlmann et al. 2021), *Monilinia laxa* (stone fruits brown rot) (Oro et al. 2014), *Geotrichum citri-auranti* (citrus sour rot) (Wang et al. 2023). Other susceptible species include *Colletotrichum gloeosporioides* in mangos (Tian et al. 2018), the grapevine pathogen *Erysiphe necator* (Lombardo et al. 2023), and the entomopathogenic *Ascosphaera apis*, which targets honeybee larvae (Iorizzo et al. 2025).

Owing to their robust antimicrobial activity and the absence of toxic by-products, numerous *M. pulcherrima* strains have been proposed as biocontrol agents for managing postharvest and soil-borne fungal pathogens. Currently, the antagonistic activity of *M. pulcherrima* is thought to be mediated by a suite of mechanisms, including the secretion of extracellular hydrolytic enzymes, such as chitinases and glucanases (Horváth et al. 2021; Mateo 2023; Minguet-Lobato et al. 2024; Pawlikowska et al. 2019; Pawlikowska and Kręgiel 2017; Steglińska et al. 2022; Tian et al. 2018). Furthermore biofilm formation, which facilitates effective niche colonization (Lapinskaitė et al. 2025; Liu et al. 2025), and the secretion of volatile organic compounds (VOCs) (Li et al. 2025; Oro et al. 2018; Parafati et al. 2015; Steglińska et al. 2022; Vepštaitė-Monstavičė et al. 2025). *Metschnikowia* trigger host defence responses via an oxidative burst (Hershkovitz et al. 2013), and outcompete pathogens for space and nutrients (Tian et al. 2018). A critical component of this competition is iron sequestration mediated by the production of pulcherriminic acid (PA), which depletes bioavailable iron in the environment and irreversibly forms pulcherrimin (Sipiczki 2006, 2020). The iron depletion process serves as a sophisticated mechanism for competitive exclusion, granting the yeast a decisive advantage by restricting the growth of rival microorganisms within the niche. Analogous to the pathways characterised in *Bacillus subtilis* and *Kluyveromyces lactis* (Krause et al. 2018; Randazzo et al. 2016), pulcherrimin biosynthesis in *M. pulcherrima* involves two enzymatic steps followed by a spontaneous reaction. The *PUL* gene cluster comprises four key components: *PUL1*, which catalyzes the formation of cyclo(L-Leu-L-Leu) from leucyl-tRNA; *PUL2*, responsible for converting cyclo(L-Leu-L-Leu) into pulcherriminic acid; *PUL3*, a putative transporter; and *PUL4*, which functions as a transcriptional regulator for the other cluster members. (Gore-Lloyd et al. 2019). Pulcherrimin, an iron (III) chelate of pulcherriminic acid (2,5-diisobutyl-3,6-dihydroxy-pyrazine-1,4-dioxide), is a red pigment that forms spontaneously in the presence of iron by a final non-enzymatic reaction. The most commonly observed form of pulcherrimin is an insoluble and non-diffusible iron chelate as pulcherriminic acid binds iron on both sides, leading to the formation of a polymeric compound (Kluyver et al. 1953). Pulcherrimin production is widely regarded as the primary mechanism underlying the antagonistic activity of the *M. pulcherrima* strains. Consequently, research efforts have focused on strategies to induce the overproduction of PA. It was demonstrated that growth on media with minimal glucose concentration and the addition of ferric ions significantly upregulate *PUL* gene expression, resulting in enhanced pulcherrimin yields (Rahmat et al. 2024). Beyond nutritional optimisation, pretreatment with amino acids such as arginine (Wang et al. 2022) and tryptophan (Zhang et al. 2023) proved effective in boosting PA synthesis and, by extension, biocontrol efficiency.

Phoma stem canker is a globally significant disease affecting oilseed rape (*Brassica napus*) and other *Brassica* species. Historically, *Leptosphaeria maculans* (current name *Plenodomus lingam*) was considered the sole causal agent. However, it was subdivided into two distinct groups: named ‘A’ (aggressive, highly virulent, Tox+) and ‘B’ (non-aggressive, weakly virulent, Tox0) (Williams and Fitt 1999). The phenotypic and genomic differences and inability of crossing between the two groups determined the distinction between two species - *L. maculans* for A-group and *L. biglobosa* for B-group (Shoemaker and Brun 2001). Globally, both species frequently co-occur within the fields and individual plants at each stage of the disease (Jacques and Balesdent 2021). In China, *L. biglobosa* is the sole causal agent of the disease, where it is associated with upper stem lesions and stem canker. In contrast, across North America, Australia, and Europe, *L. biglobosa* is regarded as a secondary pathogen, contributing significantly less to yield losses compared to the more aggressive *L. maculans* (Brazauskiene et al. 2005; Karolewski et al. 2007; Zhang et al. 2014; Zhang and Fernando 2018). *L. maculans* is capable of infecting nearly all plant tissues, including cotyledons, leaves, stems, roots, and pods, resulting in characteristic leaf lesions and stem cankers. During the infection process, the pathogen remains extracellular and exhibits a hemibiotrophic lifestyle, transitioning from biotrophy to necrotrophy within *Brassica* hosts. Among these, *Brassica napus* is the most economically significant crop. Primarily cultivated for oil production, rapeseed currently ranks third globally, following palm oil and soybean, with total production continuing to rise (FAO 2025). Worldwide, stem canker disease is estimated to cause annual losses of 5–50% (Zheng et al. 2020). The extent of yield losses is heavily contingent on weather conditions, cultivar susceptibility, fungicide efficacy, and pathogen virulence. Ongoing climate change, specifically global warming, is considered a primary driver of increased disease severity, particularly in winter oilseed rape (Brazauskiene et al. 2005). Due to its dual reproductive mode (sexual and asexual), high mutation rate, and significant gene flow, *L. maculans* readily overcomes host resistance. Consequently, stem canker outbreaks result from a complex interplay between the pathogen, host, climate, and agronomic practices, including fungicide use, resistant cultivars, crop rotation, and spatial isolation of crops. To achieve more sustainable production, an integrated management approach combining these strategies and accurate disease forecasting is essential (Vasquez‑Teuber et al. 2024). Given the extended vegetation period and high pathogen pressure, chemical fungicide protection remains a standard practice in most European rapeseed management programs. Azole-based compounds represent the predominant class of antifungals utilised in agriculture. However, the rise in global temperatures, coupled with intensive practices such as monoculture and the excessive application of azole pesticides, has led to a significant increase in azole resistance (Sanseverino et al. 2025; Verweij et al. 2020). As a result, traditional chemical interventions are becoming less effective against evolving fungal pathogens. In parallel, the European Commission’s ‘Farm to Fork’ strategy, a core component of the Green Deal, stipulates a halving of pesticide reliance by 2030 (European Commission, 2020). Such regulatory shifts create an urgent demand for sustainable biological alternatives for in-field control of major diseases such as phoma stem canker. Recent research into biocontrol strategies against *L. maculans* has explored the potential of interspecific competition within the *Leptosphaeria* genus. For instance, inoculation with the less virulent *L. biglobosa* can prime the resistance of canola plants against the highly virulent *L. maculans* (Padmathilake and Fernando 2022). However, this growth inhibition appears to be highly context-dependent, often requiring specific conditions, such as co-inoculation at the same site with equal inoculum density, that are unlikely to occur under field conditions, where both species typically complete their life cycles concurrently (Gay et al. 2023). Furthermore, the timing of arrival is critical; *L. biglobosa* can suppress the growth of *L. maculans* and inhibit the production of the phytotoxin sirodesmin PL, provided it is inoculated either before or within three days of the pathogen (Bingol et al. 2024). In addition to fungal antagonism, bacterial endophyte communities have shown promising results in reducing phoma stem canker and increasing oilseed rape yields (Schmidt et al. 2021).

Within our department, initial screening for yeast antagonism identified pulcherrimin-producing strains as potent inhibitors of *L. maculans*. While current research largely focuses on the application of *M. pulcherrima* within closed postharvest storage systems, we argue that its potential for open-field biocontrol should not be overlooked. Beyond their broad-spectrum antifungal activity and biosafety, *M. pulcherrima* strains exhibit remarkable resilience to harsh environmental conditions. This potential is supported by recent evidence where *M. pulcherrima* MPR3 application in open-field vineyards significantly reduced the incidence of powdery mildew (*E. necator*) and grey mould (*B. cinerea*), even outperforming standard organic treatments (Lombardo et al. 2023). Gaining deeper insight into the regulation of these antagonistic responses is crucial for optimising field-based biocontrol applications. Therefore, this study aimed to evaluate the inhibitory capacity of a selected *M. pulcherrima* strain against *L. maculans* and elucidate the underlying mechanisms of antagonism using diverse in vitro culture techniques. As PA synthesis is widely regarded as the primary antagonistic mechanism of these yeasts, we investigated the regulation of PA biosynthesis by analysing the expression pattern of the *PUL* gene cluster during co-culture with *L. maculans*.

## Materials and methods

### Biological material

#### Yeast isolates

Biological samples were collected at harvest from domestic orchards where no chemical plant protection products had been applied. The samples were transported in sterile bags and stored at -20 °C until analysis. Under aseptic conditions, 10 g of material (representing specific cultivars and sites) was homogenised in 90 mL of sterile saline (0.85% NaCl) using a stomacher. A series of decimal dilutions was prepared, and aliquots were spread-plated onto YGC agar pH 6.6: glucose, 20; yeast extract, 5; chloramphenicol, 0.1 and agar, 15 (g L^-1^) (BTL, Łódź, Poland). Following incubation at 28 °C, individual colonies were subcultured onto fresh YGC medium for 48 h to ensure purity. To screen for putative pulcherrimin-producing strains, isolates were transferred to YGC medium supplemented with iron(III) chloride at a concentration of 0.015 mg mL^-1^. The isolates were subjected to preliminary identification via MALDI-TOF MS, while the taxonomic classification of the strongly antagonistic strains was verified by Sanger sequencing. Finally, glycerol stocks (30% v/v) were prepared and stored at -80 °C for long-term preservation.

#### *Leptosphaeria maculans* isolate

The *L. maculans* isolate used in this study was sourced from the Institute of Plant Protection – National Research Institute in Poznań, Poland (collection number ID 2283). This strain was originally isolated from *Brassica napus*.

### Yeast species identification methods

#### Identification using MALDI-TOF mass spectrometry

Mass spectrometry analysis was performed at the Microbiological Laboratory of the Jagiellonian Center of Innovation (Kraków, Poland). A Microflex LT MALDI-TOF mass spectrometer (Bruker Daltonics, Bremen, Germany) was employed for matrix-assisted laser desorption/ionization time-of-flight analysis. Microorganisms were identified using the MALDI Biotyper system (Bruker Daltonics), supported by a regularly updated database comprising approximately 5,000 species of bacteria, yeast-like fungi, filamentous fungi, and dermatophytes. The confidence of identification was expressed as an Identification indicator (*Ii*). *Ii* ≥ 2.00 indicated high-confidence species identification, *Ii* between 1.70 and 1.99 represented low-confidence identification, and *Ii* < 1.70 were considered insufficient for reliable species-level identification.

#### Total genomic DNA isolation

Prior to genomic DNA extraction, all strains were subcultured on YPD agar: yeast extract, 10 (Biomaxima, Lublin, Poland); bactopeptone, 20 (BTL, Łódź, Poland); glucose, 20 (POCh, Gliwice, Poland) and agar, 15 (BTL) (g L^-1^), for 22 h at 28 °C. The biomass was harvested using a 10 µL loop, resuspended in 0.5 mL of saline solution (0.9% w/v NaCl), and pelleted by centrifugation. DNA extraction was performed using the Genomic Mini AX Yeast Spin Kit (A&A Biotechnology, Gdynia, Poland) according to the manufacturer’s instructions, including the optional enzymatic lysis step with lyticase at 30 °C, followed by vigorous vortexing.

DNA concentration and purity were determined using a NanoDrop ND-1000 UV spectrophotometer (Thermo Fisher Scientific, Wilmington, USA). The ratios, reflecting DNA purity, ranged between 1.9 and 2.0. DNA integrity was verified via agarose gel electrophoresis according to standard protocols (Sambrook and Russell 2001).

#### Sanger DNA sequencing and sequencing data analysis

The isolated DNA served as a template for PCR amplification using the primer pair ITS4 and NS3 (White et al., 1990). The target region encompassed the 5.8 S rDNA, two internal transcribed spacers (ITS1 and ITS2), and a fragment of the 18 S rDNA, yielding an amplicon of approximately 1.8 kb. Thermocycling conditions were as follows: initial denaturation at 95 °C for 5 min; followed by 35 cycles of 95 °C for 30 s (denaturation), 60 °C for 30 s (annealing), and 72 °C for 2 min (extension); with a final extension at 72 °C for 5 min. The resulting amplicons were resolved via agarose gel electrophoresis and purified using the Gel-Out kit (A&A Biotechnology, Gdynia, Poland) according to the manufacturer’s instructions. Sequencing was performed by Genomed S.A. (Warszawa, Poland) in both directions. The raw sequencing data were manually curated using BioEdit v7.2.5 (Ibis Biosciences, Carlsbad, CA, USA). Since the obtained sequences were too short to generate a consensus sequence, the ITS4 and NS3 sequences were subjected to BLASTN analysis against the NCBI GenBank nucleotide database (Altschul et al. 1990). The taxonomic classification of the isolates was determined based on the highest query coverage and identity coefficients obtained for the target sequence.

### Evaluation of yeast antagonistic activity

#### Plate assay with yeast on indicator

Yeast isolates were subcultured on fresh PDA medium (Potato Dextrose Agar, Liofilchem, Roseto degli Abruzzi, Italy) for 24 h at 28 °C. To prepare the liquid inoculum, biomass from each isolate was transferred into 15 mL Falcon tubes containing 3 mL of PDB medium. The cultures were incubated in duplicate for 24 h at 28 °C with constant agitation at 250 RPM using an ES-20 Orbital Shaker-incubator (Biosan). The antagonism assay was performed using the pour-plate technique. Petri dishes were prepared with PDA or PDA supplemented with iron(III) chloride at a concentration of 0.015 mg mL-1, both containing a 10% (v/v) suspension of a 72-hour-old liquid culture of *L. maculans*. Each plate was then inoculated with 10 µL of the yeast culture in two technical replicates. Control samples, consisting of the pathogen grown without yeast, were prepared simultaneously. After 7 days of incubation at 28 °C, the diameter of the fungal growth inhibition zones was measured and recorded.

#### Plate assay with indicator on yeast

A fresh yeast suspension was prepared by inoculating 20 mL of PDB medium in a 50 mL Falcon tube, followed by incubation for 24 h at 28 °C with constant agitation at 250 RPM (ES-20 Orbital Shaker-incubator, Biosan). The cell concentration was determined using a CellDrop FL automated cell counter (DeNovix Inc., Wilmington, USA). Based on these measurements, specific volumes of the culture were harvested to achieve a final concentration of 10^6^ cells mL^-1^ in the PDA medium. The cells were pelleted by centrifugation (10 min, 4,500 rpm; Multifuge^®^ 1 L-R, Thermo Scientific) and resuspended in 1 mL of sterile saline. A series of decimal dilutions was then performed to obtain final concentrations of 10^5^, 10^4^ and 10^3^ cells mL^-1^. These suspensions were incorporated into liquid PDA medium and poured into Petri dishes. Once the media solidified, a central well was created in each dish using a sterile cork borer. A mycelial plug with a diameter of 12 mm was cut from the margin of a 7-day-old *L. maculans* solid culture and placed into each well. Control plates without yeast were prepared simultaneously. After 7 days of incubation at 28 °C, the diameter of the mycelial growth was measured. The Mould Growth Inhibition Index (*Ri*) was calculated based on the arithmetic mean of two biological and two technical replicates using the following formula:

1 $$Ri\;=\;\frac{Di\;-\;Dy}{Di}\;\times\;100$$

where *Ri* is the mycelial growth inhibition index (%), *Di* is the diameter of the mycelium in the control group (mm), and *Dy* is the diameter of the mycelium in the presence of yeast (mm).

#### Double-plate assay

Yeast plates with varying cell concentrations (10^3^ – 10^6^ cells mL^-1^) were prepared as described in Section ‘Plate assay with indicator on yeast’. Separately, PDA plates were inoculated centrally with a mycelial plug with a diameter of 12 mm cut from the margin of a 7-day-old *L. maculans* solid culture. The lid of each plate was removed, and the bottom containing the yeast culture was inverted and placed over the bottom containing the mycelial plug. The two bottoms were tightly sealed with Parafilm to limit gas exchange. Control samples were prepared by joining a PDA plate containing a mycelial plug (12 mm diameter) with a yeast-free PDA plate. Additional control samples were established using two PDA Petri dishes inoculated with a mycelial plug (12 mm diameter) to investigate the impact of limited gas exchange on mould radial growth. The assemblies were incubated for 7 days at 28 °C. Following incubation, the diameters of the mycelial growth zones were measured, and the Mould Growth Inhibition Index (*Ri*) was calculated according to the formula (1).

#### Liquid co-culture assay

The yeast was subcultured on fresh YPD agar and incubated for 24 h at 28 °C. A 1 µL loop of biomass was used to inoculate 20 mL of PDB medium in 50 mL flasks, followed by incubation for 24 h at 28 °C with shaking at 250 rpm. The cell concentration was determined using a CellDrop FL automated cell counter (DeNovix Inc., Wilmington, USA). Based on these measurements, a calculated volume of the culture was centrifuged, and the resulting pellet was resuspended in fresh PDB to achieve an initial cell density of 10^6^ cells mL^-1^. For the co-culture experiments, 10 mL of this suspension was transferred to either 50 mL Falcon tubes or Petri dishes. Two mycelial agar plugs with a diameter of 12 mm cut from the margin of a 7-day-old *L. maculans* solid culture were then added to each yeast suspension. The additional plugs were placed on PDA Petri dishes to confirm the viability of mycelium. Three replicates were prepared for both treatment and control groups. The co-cultures in Petri dishes were incubated statically for 5 days at 28 °C, while those in flasks were incubated under the same conditions with continuous shaking at 200 rpm.

### Submerged co-cultivation for gene expression analysis

#### Comparison of *M. pulcherrima* growth kinetics in various culture media

The yeast strain was subcultured on fresh YPD agar and incubated for 24 h at 28 °C. A 10 µL loop of biomass was used to inoculate 10 mL of YPD, YMA: (yeast extract, 3 (Biomaxima); glucose, 10 (POCh); meat peptone (Merck, Darmstadt, Germany), 5; malt extract, 3 (Merck) and agar, 15 (BTL) (g L^-1^) ), or PDB (Potato Dextrose Broth, BTL) media in 50 mL Falcon tubes. Following incubation, the yeast suspensions were standardised to an initial *OD*_600_ of 0.1 in the respective media. Aliquots (200 µL) of the standardised cultures were dispensed into the wells of a 96-well microplate. Each condition was tested in triplicate, while uninoculated media were placed in adjacent wells to serve as negative controls and to monitor for potential cross-contamination. The plates were incubated at 28 °C for 24 h. Absorbance at 600 nm was measured every 60 min using a Multiskan Sky Microplate Spectrophotometer (Thermo Fisher Scientific, Waltham, USA), with a brief pulse-shaking step preceding each measurement. The experiment was performed in two independent biological replicates.

#### Submerged co-cultures

Yeast strain was subcultured on fresh PDA medium and incubated for 24 h at 28 °C. A 10 µL loop of biomass was used to inoculate 20 mL of PDB in 50 mL Falcon tubes. Cell concentration was determined using a CellDrop FL automated cell counter (DeNovix Inc., Wilmington, USA). Subsequently, a calculated volume of the culture was centrifuged, and the pellet was resuspended in fresh PDB to achieve an initial cell density of 10^6^ cells mL^-1^. For the co-culture experiments, three mycelial plugs with a diameter of 12 mm cut from the margin of a 7-day-old *L. maculans* solid culture were added to the yeast suspension. The additional plugs were placed on PDA Petri dishes to confirm the viability of mycelium. Axenic yeast cultures, yeast-mould co-cultures, and controls were maintained in 50 mL of medium in baffled flasks, each in biological triplicate. At 0, 24, 48, and 72 h, 1 mL aliquots were collected for: (i) *OD*_600_ measurement; (ii) spot assays (stored at -20 °C); and (iii) RNA isolation. Samples for RNA isolation were centrifuged (3 min at 4,600 rpm; MiniSpin^®^,Eppendorf), the supernatant was discarded, and the resulting pellet was resuspended in 800 µL of Fenozol for stabilisation (A&A Biotechnology, Gdynia, Poland). These samples were then stored at -80 °C until further analysis.

#### Spot assay to assess changes in yeast abundance

1 mL aliquots (obtained as described in Section ‘Submerged co-cultures’) were centrifuged for 3 min at 4,600 rpm (MiniSpin^®^,Eppendorf). The supernatant was discarded, and the cell pellets were resuspended in 1 mL of saline solution. For the assay, 200 µL of each suspension was loaded into the first column of a 96-well plate. A serial tenfold dilution was performed by transferring 20 µL of the suspension into 180 µL of sterile saline in subsequent wells. The diluted samples were then spotted onto YGC agar plates using a replica plater. The plates were incubated at 28 °C for 48 h before visual assessment.

### Reverse transcription – quantitative polymerase chain reaction (RT - qPCR)

#### Target gene selection, design and validation of the primers

The target genes and their corresponding primer sequences are listed in Table 1. Primers were designed using the Primer3-BLAST tool and synthesised by Genomed S.A. (Warszawa, Poland). To ensure broad applicability within the *M. pulcherrima* and avoid regions with high single-nucleotide polymorphism (SNP) frequency, multiple sequence alignments were performed using NCBI tools. This approach allowed for the identification of conserved regions, accounting for the intragroup heterogeneity described by Sipiczki (2024). Amplification efficiency of each primer pair was determined based on a four-point standard curve. The standardising samples were prepared as a two-fold dilution series of mixed cDNA obtained from biological replicates of co-cultures B. Each standardising point was amplified in duplicate.

**Table 1:** Description of the designed primer pairs. Accession numbers correspond to the sequences of *Metschnikowia pulcherrima (strain APC 1.2*, *type strain CBS 5833T)*

Gene			Primers				
Name (symbol)	Accession number	Range [bp]	Label	Start	Stop	Sequence (5’->3’)	Product length [bp]
*PUL1*	CP034458.1	1,434,770–1,436,146	MPul_PUL1_Fw	1,435,633	1,435,652	GATCTCGGAAAGGCTCTGGG	308
			MPul_PUL1_Rv	1,435,940	1,435,921	AGGGGTACTGTTTGTGCTGG	
*PUL2*	CP034458.1	1,432,720–1,434,162	MPul_PUL2_Fw	1,433,254	1,433,273	AACCAACGACCACGACATGA	303
			MPul_PUL2_Rv	1,433,556	1,433,537	GTGCCGCTCCATAGTGAGTT	
*PUL3*	CP034458.1	1,429,481–1,430,440	MPul_PUL3_Fw	1,429,634	1,429,653	GCCGTCCCCACAAAGAAAAC	254
			MPul_PUL3_Rv	1,429,887	1,429,868	TGAGGAAACACGAGACTGCC	
*PUL4*	CP034458.1	1,430,448–1,432,091	MPul_PUL4_Fw	1,431,528	1,431,547	CTCTGCTCTCACCTGCAACA	345
			MPul_PUL4_Rv	1,431,872	1,431,853	ACGCTCATATCGCCCATCAG	
transcriptional gene regulator (*SNF2)*	CP034458.1	2,549,829–2,554,565	MPul_SNF2_Fw	2,553,779	2,553,798	CGGGAGAGGAAACCGTGAAA	319
			MPul_SNF2_Rv	2,554,097	2,554,078	TGTTGCTCGCACTTCAGGAT	
6-phosphofructo-2- kinase (*PFK*)	CP034456.1	3,186,568–3,189,471	MPul_PFK_Fw	3,187,964	3,187,983	CTGCCGACTTTTTCAACCCG	315
			MPul_PFK_Rv	3,188,278	3,188,259	AGTCTCGTAAGGCCACCTCT	
actin (*ACT1*)	AJ745126.1	1–979	MPul_ACT_Fw	477	496	AGATTTTGTCCGAGCGTGGT	250
			MPul_ACT_Rv	726	707	ATACCAACAGCCTCCAAGCC	

#### RNA isolation

Total RNA was extracted from *M. pulcherrima* biomass using the Bead-Beat Total RNA Mini Kit (A&A Biotechnology, Gdynia, Poland) according to the manufacturer’s instructions. RNA concentration and purity were determined using a NanoDrop ND-1000 UV spectrophotometer (Thermo Fisher Scientific, Wilmington, USA). The ratios, reflecting RNA purity, ranged between 1.9 and 2.1. RNA integrity was verified via agarose gel electrophoresis according to standard protocols (Sambrook and Russell 2001). The observed electrophoretic patterns for all samples exhibited sharp and intense bands corresponding to the 26 S and 18 S rRNA subunits, with no visible RNA degradation.

#### Reverse transcription (RT) reaction

The amount of input RNA for the RT reaction was standardised to minimise experimental variation. First-strand cDNA synthesis was performed using 500 ng of total RNA and oligo(dT)_18_ primer in a final volume of 20 µL, employing the TranScriba™ NoGenome™ Kit (A&A Biotechnology, Gdynia, Poland). The reaction was conducted in a T100 Thermal Cycler (Bio-Rad Laboratories, Inc., Hercules, CA, USA) using a protocol that included an integrated genomic DNA removal step. The thermal profile was as follows: 42 °C for 2 min (gDNA depletion), followed by 4 °C for 10 min, 42 °C for 60 min (RT step), and 70 °C for 5 min (enzyme inactivation). To monitor for potential genomic DNA contamination in subsequent qPCR analysis, no-reverse transcriptase (RT-minus) controls were prepared using the NoGenome™ master mix and template RNA without the addition of TranScriba™ reverse transcriptase.

#### qPCR reactions

qPCR was conducted using a CFX96 Real-Time PCR Detection System (Bio-Rad Laboratories, Hercules, CA, USA). Each reaction mixture (10 µL total volume) contained 5 µL of RT PCR Mix SYBR^®^ MasterMix (A&A Biotechnology, Gdynia, Poland), 0.2 µL of each primer (10 µM forward and reverse), and 1 µL of cDNA template. The reactions were carried out in white 96-well PCR plates (Bio-Rad Laboratories) to enhance the fluorescent signal. The thermocycling conditions were as follows: initial denaturation at 95 °C for 3 min, followed by 40 cycles of 95 °C for 15 s (denaturation), 60 °C for 30 s (annealing), and 72 °C for 1 min (extension). To confirm reaction specificity, a melting curve analysis was performed from 65 to 95 °C with 0.5 °C increments every 5 s. All samples were analysed in technical duplicates, and the observed variation between replicates was negligible. To ensure the absence of contamination, a No-Template Control (NTC) was included for each primer pair. Data acquisition and analysis were performed using CFX Maestro™ Software version 1.1 (Bio-Rad Laboratories) the Bio-Rad CFX Manager software.

#### Normalised gene expression (2^-∆∆Cq^)

Gene expression analysis was performed using CFX Maestro™ Software version 1.1 (Bio-Rad Laboratories). The transcription levels of target genes (from the *PUL* gene cluster and *SNF2*) were normalised against the *ACT1* house-keeping gene as an internal control. Fold-change in gene expression was calculated using the method 2^−∆∆Cq^ (Livak and Schmittgen 2001). Axenic yeast cultures served as the calibrator group (control). A fold-change threshold of 4.0 was applied to identify biologically significant up- or down-regulation. Statistical significance was determined at *p* < 0.05.

### Statistical analysis

Statistical analysis of culture parameters was performed using Microsoft Excel (Windows 365). Data are expressed as the mean ± standard deviation (SD) of two or three biological replicates, each analysed in two technical replicates. The significance of differences between groups was determined using an unpaired, two-tailed Student’s *t*-test. Statistical significance was defined as *p* < 0.05.

## Results

### Screening and identification of yeast isolates for antagonism against *L. maculans*

**Fig. 1:**
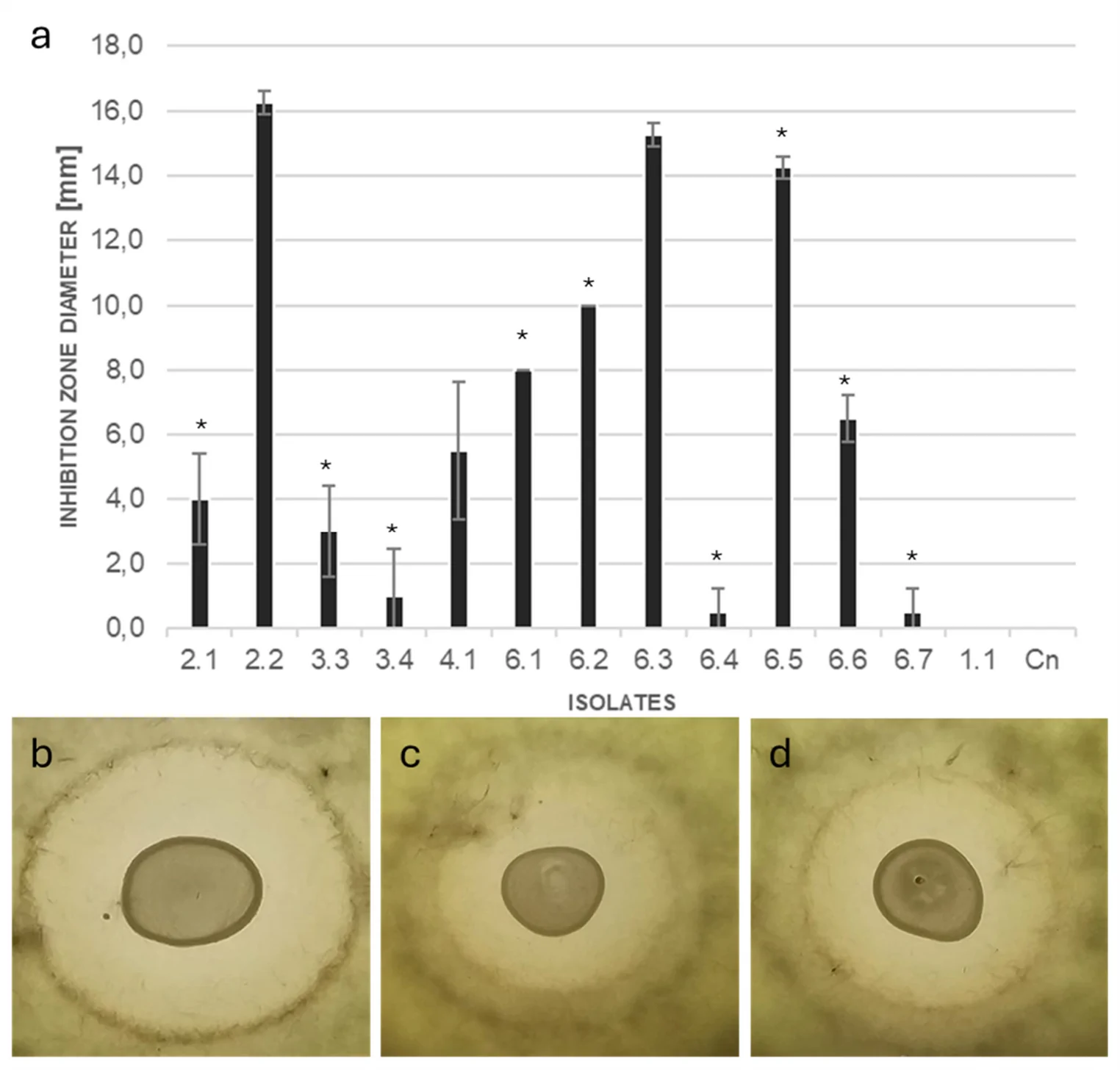
Results of the plate assay of yeast isolates on indicator (*L. maculans*). **a**. Average diameters of inhibition zones (mm) for yeast isolates compared to the negative control (Cn). Values represent means ± SD of biological and technical replicates (*n* = 2). **b**. Inhibition zone of isolate 2.2. **c**. Inhibition zone of isolate 6.3. **d**. Inhibition zone of isolate 6.5. Asterisks indicate isolates significantly different from isolate 2.2 (*p* < 0.05)

Selected isolates, particularly those producing pulcherrimin, were preliminarily identified using MALDI-TOF mass spectrometry (Table 2). Only a few isolates were assigned to a specific species with low confidence, as the identification scores (*Ii*) fell within the range of 1.70–1.99. In several cases, the scores were below 1.69, meaning no species-level identification was possible. The results of molecular identification of highly potent antagonistic isolates are presented in Table 3. Generating a consensus sequence was precluded by insufficient read lengths from individual primers. Consequently, Table 3 displays the reference alignment results for the separate sequences obtained using both ITS4 and NS3 primers. Antagonistic interactions between the yeast isolates and *L. maculans* were evaluated using the plate assay with yeast on the indicator. Figure 1a presents the average inhibition diameters of the clear area calculated from two independent experiments, each performed in technical duplicate. Statistical analysis revealed significant antagonistic activity against the mould for isolate 2.2 (*p* < 0,05) in the majority of cases. Notably, the zones of complete inhibition (affecting both aerial and substrate mycelium) for isolates 6.3 (Fig. 1c) and 6.5 (Fig. 1d), which exhibited similar inhibitory activity, were narrower than those observed for isolate 2.2 (Fig. 1b). Based on these observations, strain 2.2, isolated from *Malus domestica* fruit, was selected for further investigation of its interaction with *L. maculans*. This strain was identified based on a high alignment identity coefficient to rDNA region of *Metschnikowia pulcherrima* strain CBS 5833 (Table 3).

**Table 2:** Characteristics of the yeast isolates (origin, pigment production, and MALDI-TOF mass spectrometry identification results)

Origin	Isolate	Pigment production	Species	Identification indicator
Flowers of *Rosa damascena*	1.1	+	*Metschnikowia pulcherrima*	1,83
Fruits of *Malus domestica* ‘Yellow Transparent’	2.1	+	*Metschnikowia pulcherrima*	1,60
	2.2	+	NI	
Fruits of *Rubus idaeus* ‘Delniwa’	3.1	-	*Pichia kluyveri*	1,52
	3.2	-	NI	
	3.3	+	NI	
	3.4	+	NI	
Fruits of *Prunus domestica ‘*Herman*’*	4.1	+	*Metschnikowia pulcherrima*	1,98
Fruits of *Malus domestica* ‘Early Geneva’	6.1	+	*Metschnikowia pulcherrima*	1,94
	6.2	+	NI	
	6.3	+		
	6.4	+		
	6.5	+	*Metschnikowia pulcherrima*	1,83
	6.6	+	*Pichia kluyveri*	1,63
	6.7	+		
Fruits of *Vitis vinifera* ‘Canadice’	7.1	-	*Hanseniaspora uvarum*	2,25
	7.2	-		
	7.3	-		
	7.4	-		
	7.5	-		

**Table 3:** Molecular identification of strongly antagonistic isolates based on BLASTn analysis of the PCR-amplified rDNA region

Isolate	ITS4 Primer					NS3 Primer				
	Sequence length [BP]	Query coverage[%]	Identity coefficient[%]	E-value	Scientific name/accession	Sequence length [BP]	Query coverage[%]	Identity coefficient [%]	E-value	Scientific name/accession
2.2	405	100	93.58	5e-175	*Metschnikowia pulcherrima*/ KY104205.1	813	100	99.51	0.0	*Metschnikowia pulcherrima*/ MK394155.1
6.3	431	100	97.91	0.0	*Metschnikowia* sp./ KM243726.1	516	100	93.60	0.0	*Metschnikowia* sp./ MK945766.1
6.5	447	100	97.54	0.0	*Metschnikowia* sp. /KM243730.1	511	99	84.51	7e-146	*Metschnikowia* sp. /MK945766.1

*BP* base pair

### Assessment of the antagonistic properties of a selected *M. pulcherrima *isolate in in vitro tests

**Fig. 2:**
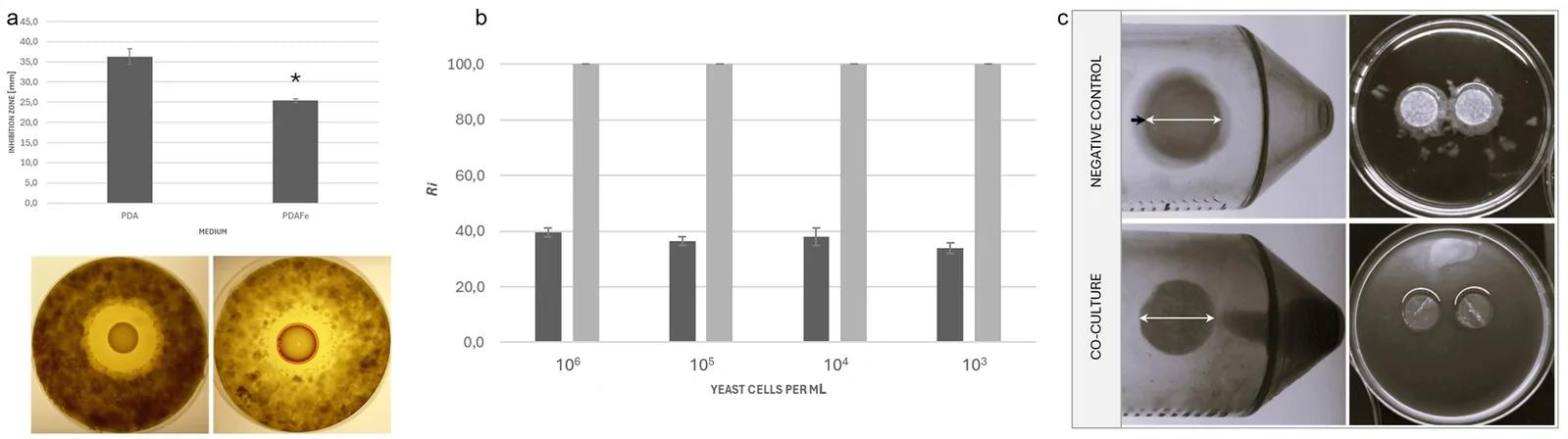
Assessment of the antagonistic properties of the selected *M. pulcherrima* strain against *L. maculans* in in vitro assays.** a**. Significant decrease in the inhibition zone diameter (mm) on PDA supplemented with iron *(*p* < 0.001). Values represent means ± SD of biological and technical replicates (*n* = 2). **b**. Comparison of the mould growth inhibition index, *Ri* (%), obtained using the indicator on yeast plate assay () and the double plate assay (). Values represent means ± SD of biological and technical replicates (*n* = 2). **c**. Results of the liquid co-culture assay conducted in 50 mL Falcon tubes and Petri dishes

An additional antagonism assay was performed on standard PDA and iron-supplemented PDA (PDA_Fe_) (0.015 mg mL^− 1^) to determine whether increased iron availability influences the antifungal effect. Iron supplementation significantly attenuated the antagonistic activity (*p* < 0.001), as shown in Fig. 2a. Representative images in Fig. 2a show that on standard PDA, the inhibition zone remains completely clear. In contrast, on PDA_Fe_ plates, the inhibition zone was significantly smaller. The antagonism of the selected *M. pulcherrima* strain was further verified using an inverse configuration (the indicator-on-yeast test). Various yeast cell densities were incorporated into the PDA medium ( 10^3^-10^6^ cells mL^⁻¹^). In this setup, which ensured direct contact between the microorganisms, complete inhibition of mould growth was observed across all tested yeast concentrations (Fig. 2b). The same cell concentrations were evaluated in a double plate assay for detecting VOCs-mediated antagonism. In this case, the mould growth inhibition approximated 40% of the total inhibitory effect (Fig. 2b). However, as the yeast density was reduced, the inhibitory effect significantly decreased compared to the 10^6^ cells mL^⁻¹^ group (*p* < 0.05). Consequently, this optimal concentration was selected for subsequent submerged co-culture experiments. As shown in Fig. 2c, the presence of yeast at an initial density of 10^6^ cells mL^− 1^ resulted in the total suppression of mould growth in both Falcon tubes and Petri dishes, compared with the negative controls, in which mycelial radial growth was observed.

A significant reduction in the mycelial radial growth was observed in the control samples of two combined PDA Petri dishes inoculated with a mycelial plug after 7 days in the double-plate assay (60 ± 2 mm vs. 72 ± 3 mm; *p* < 0.05). The confirmed restricted gas exchange could have intensified competition for oxygen between the numerically predominant, fast-growing cells of *M. pulcherrima* and *L. maculans* mycelium in the double-plate assay. Therefore, the observed high degree of mould growth inhibition does not unequivocally determine the actual effect of volatile metabolites.

### Expression of *PUL* cluster genes in *M. pulcherrima* and *L. maculans* interaction

**Fig. 3:**
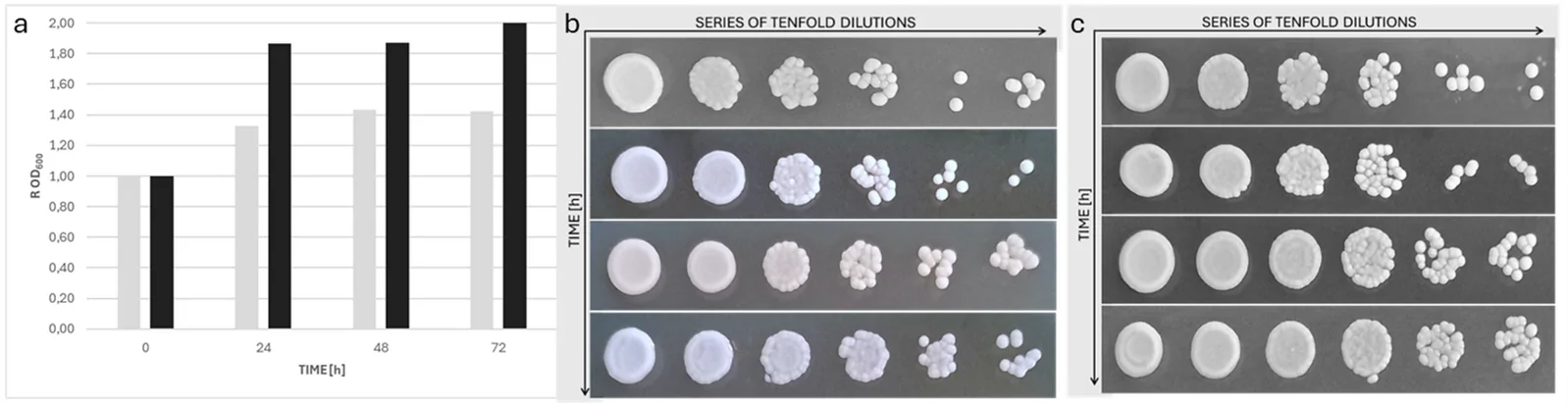
*M. pulcherrima* growth rate in submerged cultures. **a**. Relative growth rates (R _*OD*600_) of *M. pulcherrima* axenic cultures () and co-cultures with *L. maculans* (■). **b**,** c**. Spot assay presenting changes in yeast abundance. Serial ten-fold dilutions (indicated by horizontal arrows) of yeast axenic culture (**b**) and co-cultures (**c**) at 0, 24, 48 and 72 h (indicated by vertical arrows)

No statistically significant differences in absolute *OD*_600_ were observed between axenic yeast cultures and yeast-mould co-cultures over the 24–72 h period (*p* > 0.05). However, the relative growth rates (calculated against initial *OD*_600_ values) differed significantly. While the absorbance of the axenic reference culture increased by 50%, that of the co-cultures increased by 100% (Fig. 3a). Accurate *OD*_600_ readings were hindered by the adsorption of yeast cells onto starch chains present in the PDB medium, which resulted in highly heterogeneous suspensions. To address this, the cultures were evaluated using a spot assay, which confirmed yeast viability throughout the co-cultivation period and demonstrated that yeast proliferated faster in the presence of the mould than in the axenic control (Fig. 3b, c). Microscopic examination of wet mounts at successive time points revealed no fungal morphological features, confirming the total inhibition of *L. maculans* (Fig.S1). The growth kinetics of the *M. pulcherrima* strain in PDB were compared to standard YPD and YMA media using a microplate format (Fig.S2). No significant differences in growth were found among the tested media. The Pearson correlation coefficients for growth curves in PDB versus YPD and YMA were 0.998 and 0.985, respectively, confirming that PDB is a suitable medium for these studies. 

**Fig. 4:**
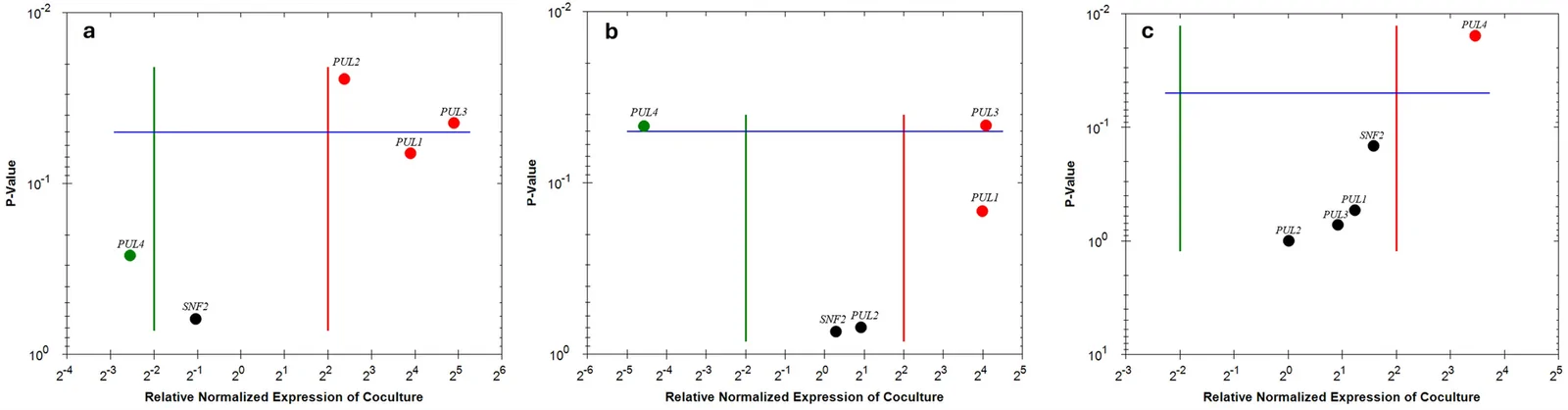
Volcano plots of relative normalised gene expression in co-cultures compared to axenic controls at 24 h (**a**), 48 h (**b**), and 72 h (**c**). The fold-change threshold is set at 4.0. Upregulated targets are indicated by (), downregulated targets by (), and non-significant changes by (●). The horizontal blue line represents the significance threshold *p* < 0.05

At 24 h of co-cultivation with *L. maculans*, a significant upregulation of the *PUL2* and *PUL3* genes was observed in *M. pulcherrima*, with fold-changes of 5.19 and 29.73, respectively (*p* < 0.05, Fig. 4a). Although *PUL1* exhibited a 14.89-fold increase, the *p*-value (0.0664) slightly exceeded the significance threshold. No significant changes were recorded for *PUL4* and *SNF2* (fold-change < 4.0). By the second day (48 h), *PUL3* remained significantly upregulated (16.86-fold, *p* < 0.05). In contrast, *PUL1* showed a 15.82-fold increase that did not reach statistical significance (Fig. 4b). Notably, a significant downregulation was observed for *PUL4* (**-**23.94-fold, *p* < 0.05), while changes for *PUL2* and *SNF2* were non-significant. On the third day (72 h), *PUL4* expression shifted to a significantly elevated level (10.98-fold, *p* < 0.05, Fig. 4c). Conversely, a downward trend in expression compared to the axenic control was observed for *PUL1*,* PUL2*,* PUL3*, and *SNF2*, though these results were not statistically significant. Normalising against an alternative reference gene, *PFK*, did not resolve the observed fluctuations. The *PFK* primer pair exhibited low amplification efficiency and late C_q_ values, resulting in poor technical reproducibility. Consequently, it was excluded from the normalisation process (Fig.S3). The lack of statistical significance in several instances was attributed to high inter-replicate variability across biological replicates, which masked the observed trends in fold-change. 

## Discussion

The MALDI-TOF mass spectrometry identification scores yielded values indicating either a low probability of species affiliation or precluding taxonomic classification of the pigment-producing environmental isolates. These results likely reflect the phenotypic variability of *M. pulcherrima* strains. This is consistent with the findings of Sipiczki (2022), who detailed the internal diversity of the D1/D2 and ITS regions, noting that dimorphisms, single-nucleotide polymorphisms (SNPs), and variations in repeat numbers are common in this species. Additionally, recent genomic analyses have revealed varying copy numbers and diverse structural arrangements of the genes responsible for the production and transport of pulcherriminic acid (the *PUL* cluster) among different *M. pulcherrima* strains (Larini et al. 2025). Such intraspecies genetic variability may determine phenotypic plasticity, which is hypothesised to be a key component of adaptive responses (Quevedo-Caraballo and Álvarez-Pérez 2025). *M. pulcherrima* strains, often acting as generalists in floral nectar, exhibit reversible stochastic epigenetic-like silencing of pulcherrimin production (Sipiczki and Czentye 2024). Given that floral nectar is a highly variable microenvironment, this stochasticity likely promotes intra-population trait variation. This is visually evident in *M. pulcherrima* colonies, which frequently display sectored pigmentation due to differential pigment secretion (Sipiczki et al. 2024). Furthermore, the fact that heavily pigmented clones exhibit superior inhibitory activity compared to non-pigmented ones directly links this phenotypic variability to the success of interspecies interactions and biocontrol efficacy. This stochasticity may explain the high inter-replicate variability observed in our expression analysis of the *PUL* genes.

Preliminary analysis of the antagonistic properties of various yeast isolates against *L. maculans* led to the selection of strain 2.2 classified as *M. pulcherrima*, as the most effective. Supplementing the medium with iron, at concentrations typically used in selective media for pulcherrimin detection, significantly attenuated the antagonistic effect. This indirectly demonstrates that the PA biosynthetic pathway plays a crucial role in limiting fungal growth within this interaction. Naturally occurring pigmentless mutants of *M. pulcherrima*, characterised by a premature stop codon in the chromatin regulator gene *SNF2*, exhibited reduced antagonistic activity against pathogens such as *Botrytis caroliniana* (Gore-Lloyd et al. 2019). RNA-seq data comparison between wild-type and *snf2* mutant cells confirmed broad transcriptional changes, with the *PUL* genes among the most strongly downregulated transcripts. Furthermore, most genes encoding secreted proteases and various transporters were significantly less transcribed in the mutant after 5–6 h of culture in PDB. These transcriptomic fluctuations suggest that the reduced antifungal activity of the *snf2* mutant results not only from the absence of pulcherrimin but also from altered levels of multiple factors contributing to the complex biocontrol phenotype. Sipiczki and Czentye (2024) concluded that transitions between the active and silent states of PA synthesis correlate with the antimicrobial antagonism states. This correlation reinforces the theory that pulcherrimin formation and iron depletion are the primary mechanisms of biocontrol in *Metschnikowia* spp. Moreover, *PUL* deletion studies in other *Metschnikowia* strains resulted in a loss of inhibition against *Geotrichum citri-aurantii* (Wang et al. 2022) and *Botrytis cinerea* (Freimoser et al. 2024). Recent research into fully functional heterologous pulcherriminic acid production further replicates this iron-dependent growth inhibition (Valero et al. 2025). Collectively, these genetic engineering studies demonstrate that the pulcherrimin production mechanism alone may be sufficient to drive yeast antagonism.

The reduction of yeast antagonism in the presence of elevated iron concentrations has been widely reported. Sipiczki (2006) observed that iron supplementation led to a decrease in the inhibition of *B. cinerea* growth. Similarly, Wang et al. (2023) demonstrated reduced inhibition of citrus fruit spoilage moulds, *P. digitatum*, *P. italicum*, and *G. citri-aurantii*, under increased iron levels. In those studies, the size of inhibition zones decreased to zero as the concentration of Fe^3+^ increased. Notably, while iron concentrations did not significantly affect the production of PA by the yeast, they resulted in a gradual reduction in the width of the pigment halos. In vivo experiments on citrus fruits mirrored these in vitro findings. However, even with high iron concentrations added to fruit wounds, *M. pulcherrima* did not completely lose its effectiveness against moulds. This suggests that the biocontrol of citrus diseases by pulcherrimin-producing *Metschnikowia* species involves multiple mechanisms, among which iron competition plays a pivotal role. Other studies confirmed that the localisation and intensity of the pigmented zone were strongly dependent on the concentration of iron cations. Higher concentrations resulted in darker and narrower rings (Horváth et al. 2021). PA secreted into the extracellular space sequesters available iron to form stable pulcherrimin near the yeast colonies. At high iron concentrations, PA may not be sufficient to induce a total deficiency in the wider environment, thus reducing its deleterious effect on surrounding microorganisms. Instead, pulcherrimin binds excessive iron, restricting its distribution and potentially protecting yeast cells against iron overload. Observations by Mažeika and coworkers (2021) and Melvydas and coworkers (2025) revealed that cells involved in pulcherrimin accumulation undergo significant changes, eventually transforming into red, iron-rich chlamydospores. Under conditions of extreme iron overload, these chlamydospores accumulate excessive pulcherrimin, ultimately leading to loss of viability and decay. These findings highlight the exceptional phenotypic plasticity of *M. pulcherrima* in modulating the PA biosynthetic pathway in response to environmental shifts, further supporting its potential as a robust biocontrol agent.

In submerged co-cultures of *L. maculans* and *M. pulcherrima*, complete inhibition of mould growth was observed at the selected yeast concentration. Similarly, no growth of other phytopathogens (*A. alternata*,* R. solani*,* B. cinerea*, and *M. laxa*) was recorded in co-cultures with *M. pulcherrima* (Perek et al. 2025). Türkel and coworkers (2014) also demonstrated the total suppression of pathogens, including *Penicillium expansum*, *Fusarium* sp., and *Rhizopus* sp. in grape juice. Aragno and coworkers (2024) showed similar results by co-culturing *Metschnikowia* yeast with the bacterium *Gluconobacter oxydans* and the yeast *Brettanomyces bruxellensis* in grape juice. The complete inhibition observed in submerged cultures mirrors the results of direct contact assays, where a uniform distribution of yeast cells was employed. This indicates that the inhibitory effect is cell-density dependent, requiring a specific threshold of yeast cells to be effective. For instance, the initial concentration of *M. pulcherrima* strains remained a critical factor for the effective control of *P. expansum* and the reduction of patulin accumulation (Settier-Ramírez et al. 2021). Furthermore, Steglińska and coworkers (2022) reported that culture filtrates devoid of yeast cells exhibited no antimicrobial activity, confirming that viable cells are essential for biocontrol. This suggests that antagonistic mechanisms may be induced or triggered by the presence of competing microorganisms.

The significant mycelial growth inhibition observed in the presented double-dish assay suggests a potential contribution of volatile organic compounds (VOCs) to the antagonistic activity of *M. pulcherrima*. The inhibition effect may also be triggered by oxygen competition between the yeast and the aerobic mould *L. maculans*, due to the restricted air volume characteristic of the double-dish methodology (Sipiczki 2023). Notably, the volatile compound identified in *M. pulcherrima* monocultures was dihydrojasmonic acid, a derivative of jasmonic acid, a crucial plant hormone that regulates stress responses by activating defence pathways (Perek et al. 2025). Moreover, VOCs production has been identified as a key mechanism against *B. cinerea* (Parafati et al. 2015) as well as *P. digitatum* and *P. italicum* (Parafati et al. 2016). Unlike yeast chitinases, which require direct contact, VOCs exhibit high diffusivity. They can spread through the atmosphere and porous soil or plant structures, acting multi-directionally within the tripartite interaction. Recently, *M. pulcherrima* was also found effective against *Ascosphaera apis*, the causative agent of chalkbrood disease in bees. This antifungal activity was attributed to nutrient competition, PA secretion, and strain-specific VOC profiles (Iorizzo et al. 2025). The importance of strain-specificity is further supported by studies showing distinct variations in antimicrobial properties against *B. cinerea* among different strains of the same species (Liu et al. 2025).

In the submerged co-cultures used in the present study, we observed accelerated yeast growth compared to axenic cultures. Simultaneously, the growth of the mycelium, confined within the agar disc, was inhibited, in contrast to the negative controls, where the mycelium spread markedly. This co-culture design restricted the mould’s access to nutrients and space, which the yeast distributed throughout the medium utilised efficiently. Given that the growth conditions were otherwise identical, these observations suggest a link between increased yeast proliferation and the presence of the mould. The increased proliferation of yeast might be triggered by a metabolic response to chemical signals emitted by the *L. maculans* inoculum, or by hormetic stress induced by mould metabolites at sub-inhibitory concentrations. Comparative expression analysis showed that the transcription of the *PUL* cluster genes was modulated in the presence of the competitor, representing an adaptive, inducible defence mechanism. The significant upregulation of *PUL3* and *PUL2* within 24 h confirms that PA is a critical factor in the antagonism against the mould, likely induced in the initial stages of the interaction following the perception of signals from the competitor. At 48 h, *PUL3* (the transporter of PA and pulcherrimin) was significantly upregulated, while *PUL4* expression remained significantly reduced. However, by 72 h, upregulation was limited solely to *PUL4*, the gene identified as a transcription factor for *PUL1*,* PUL2*, and *PUL3* (Krause et al. 2018). The statistically significant increase in *PUL4* expression, alongside the downregulation trend of other genes in the cluster during the stationary phase, suggests that this transcription factor may act as a repressor of the PA biosynthesis and transport pathway. The *PUL4* product in yeast may be a functional analogue of the *yvmB* gene, which acts as a repressor of the PA biosynthesis operon in *B. subtilis* (Randazzo et al. 2016). In *B. subtilis*, *yvmB* expression increases upon entry into the stationary phase, indicating that iron deficiency induces its expression. Furthermore, *yvmB*-deletion mutants overproduce red pigment in iron-rich media. The repressor nature of *PUL4* is further supported by findings where both *PUL3* (mediating iron-pulcherrimin uptake) and *PUL4* were significantly upregulated in media using pulcherrimin as the sole iron source (Wang et al. 2025). We observed no significant changes in *SNF2* expression levels during the co-culture period. While *SNF2* is known to regulate PA biosynthesis (Gore-Lloyd et al. 2019), its role as a chromatin remodeler and the global transcriptomic changes associated with its deletion suggest its regulatory function is likely non-specific. This is consistent with studies on *M. citriensis*, where *PUL1* and *PUL2* expression increased gradually over time, while *SNF2* levels remained unchanged, further indicating that *SNF2*-mediated regulation of the *PUL* pathway is, at the very least, indirect (Zhang et al. 2023).

## Conclusions

Pulcherrimin-producing environmental strains isolated from organically grown fruit exhibited significant in vitro antagonistic activity against *L. maculans*. The selected isolate, which exhibited notable antagonistic potential and was identified as *M. pulcherrima*, inhibited mould growth in in vitro tests. The isolate reduced antagonism under iron supplementation, and the analysis of *PUL* gene expression during co-culture with *L. maculans* supported an important role for the PA biosynthesis pathway in inhibiting pathogen growth. The study reveals a clear temporal regulation of the *PUL* cluster: initial induction of *PUL1*,* PUL2* and *PUL3* at the onset of co-culture, followed by the upregulation of *PUL3*, and finally, the presumed shutdown of the PA pathway via *PUL4*-mediated repression as iron reserves are depleted. The dominance of yeast in submerged co-cultures provides a robust system for investigating the molecular basis of yeast antagonism induced by fungal competitors. Future research should focus on a detailed analysis of *PUL* cluster genes expression regulation under selected environmental conditions, as well as comprehensive volatilome profiling and targeted Ehrlich Pathway gene expression analysis within the co-culture system. An in-depth understanding of the multiple mechanisms governing *M. pulcherrima* antagonism against *L. maculans* will facilitate the identification of factors that enhance the yeast’s potential to counteract fungal growth. 

## Supplementary Information

Below is the link to the electronic supplementary material.

Supplementary Material 1 (DOCX 624 KB)

Supplementary Material 2 (DOCX 23.9 KB)

Supplementary Material 3 (DOCX 64.6 KB)

## Data Availability

The datasets generated during the current study are available from the corresponding author upon request.
