# Supplementary material for: Antagonism-driven upregulation of the pulcherriminic acid biosynthetic pathway in Metschnikowia pulcherrima and Leptosphaeria maculans interaction
Source: World J Microbiol Biotechnol. 2026 Aug 4;42(8):444. doi: 10.1007/s11274-026-05168-6 (PMC13437667; doi:10.1007/s11274-026-05168-6)
Supplement: Supplementary file 2 [file 11274_2026_5168_MOESM2_ESM.docx]

Antagonism-driven upregulation of the pulcherriminic acid biosynthetic pathway in *Metschnikowia pulcherrima* and *Leptosphaeria maculans* interaction

World Journal of Microbiology and Biotechnology

Borkowska Monika*, Wita Agnieszka, Myszka Kamila

Department of Biotechnology and Food Microbiology, Poznan University of Life Sciences, ul. Wojska Polskiego 48, 60-637 Poznan, Poland

*Corresponding Author: [monika.borkowska@up.poznan.pl](mailto:monika.borkowska@up.poznan.pl)

**Figure S2 *M. pulcherrima* growth kinetics in YPD (**◊**), PDB (**□**) and YM (**∆**) media.** Microplate minicultures were incubated at 28 ℃ for 24 h. Absorbance was measured every 60 min at 600 nm. Values indicate means ± SD of technical replicates (n = 3).
