# Supplementary material for: Antagonism-driven upregulation of the pulcherriminic acid biosynthetic pathway in Metschnikowia pulcherrima and Leptosphaeria maculans interaction
Source: World J Microbiol Biotechnol. 2026 Aug 4;42(8):444. doi: 10.1007/s11274-026-05168-6 (PMC13437667; doi:10.1007/s11274-026-05168-6)
Supplement: Supplementary file 3 [file 11274_2026_5168_MOESM3_ESM.docx]

Antagonism-driven upregulation of the pulcherriminic acid biosynthetic pathway in *Metschnikowia pulcherrima* and *Leptosphaeria maculans* interaction

World Journal of Microbiology and Biotechnology

Borkowska Monika*, Wita Agnieszka, Myszka Kamila

Department of Biotechnology and Food Microbiology, Poznan University of Life Sciences, ul. Wojska Polskiego 48, 60-637 Poznan, Poland

*Corresponding Author: [monika.borkowska@up.poznan.pl](mailto:monika.borkowska@up.poznan.pl)

**Figure S3** Standard curves for determining the amplification efficiency (E) of primer pairs targeting cDNA fragments of *PUL1*, *PUL2*, *PUL3*, *PUL4*, *ACT1*, *SNF2*, and *PFK*.
